# The impact of p53 function on the metabolic activation of the carcinogenic air pollutant 3-nitrobenzanthrone and its metabolites 3-aminobenzanthrone and *N*-hydroxy-3-aminobenzanthrone in human cells

**DOI:** 10.1093/mutage/gey025

**Published:** 2018-09-11

**Authors:** Laura E Wohak, Ann-Christin Baranski, Annette M Krais, Heinz H Schmeiser, David H Phillips, Volker M Arlt

**Affiliations:** 1 Department of Analytical, Environmental and Forensic Sciences, MRC-PHE Centre for Environment and Health, King’s College London, London, UK; 2 Section of Molecular Carcinogenesis, Institute of Cancer Research, Sutton, Surrey, UK; 3 Division of Radiopharmaceutical Chemistry, German Cancer Research Center (DKFZ), Im Neuenheimer Feld, Heidelberg, Germany; 4 NIHR Health Protection Research Unit, Health Impact of Environmental Hazards, King’s College London, Public Health England and Imperial College London, London, UK

## Abstract

The tumour suppressor p53, encoded by *TP53*, is a key player in a wide network of signalling pathways. We investigated its role in the bioactivation of the environmental carcinogen 3-nitrobenzanthrone (3-NBA)found in diesel exhaust and its metabolites 3-aminobenzanthrone (3-ABA) and *N*-hydroxy-3-aminobenzanthrone (*N*-OH-3-ABA) in a panel of isogenic human colorectal HCT116 cells differing only with respect to their *TP53* status [i.e. *TP53(+/+*), *TP53(+/−*), *TP53(−/−*), *TP53(R248W/+*) or *TP53(R248W/−*)]. As a measure of metabolic competence, DNA adduct formation was determined using ^^32^^P-postlabelling. Wild-type (WT) p53 did not affect the bioactivation of 3-NBA; no difference in DNA adduct formation was observed in *TP53(+/+*), *TP53(+/−*) and *TP53(−/−*) cells. Bioactivation of both metabolites 3-ABA and *N*-OH-3-ABA on the other hand was WT-*TP53* dependent. Lower 3-ABA- and *N*-OH-3-ABA-DNA adduct levels were found in *TP53(+/−*) and *TP53(−/−*) cells compared to *TP53(+/+*) cells, and p53’s impact was attributed to differences in cytochrome P450 (CYP) 1A1 expression for 3-ABA whereas for *N*-OH-3-ABA, an impact of this tumour suppressor on sulphotransferase (SULT) 1A1/3 expression was detected. Mutant R248W-p53 protein function was similar to or exceeded the ability of WT-p53 in activating 3-NBA and its metabolites, measured as DNA adducts. However, identification of the xenobiotic-metabolising enzyme(s) (XMEs), through which mutant-p53 regulates these responses, proved difficult to decipher. For example, although both mutant cell lines exhibited higher *CYP1A1* induction after 3-NBA treatment compared to *TP53(+/+*) cells, 3-NBA-derived DNA adduct levels were only higher in *TP53(R248W/−*) cells but not in *TP53(R248W/+*) cells. Our results show that p53’s influence on carcinogen activation depends on the agent studied and thereby on the XMEs that mediate the bioactivation of that particular compound. The phenomenon of p53 regulating *CYP1A1* expression in human cells is consistent with other recent findings; however, this is the first study highlighting the impact of p53 on sulphotransferase-mediated (i.e. SULT1A1) carcinogen metabolism in human cells.

## Introduction

Combustion-derived pollutants are released into the environment from industrial activities, traffic emissions and domestic heating. Outdoor air pollution and diesel engine exhaust have been classified as carcinogenic to humans (Group 1) by the International Agency for Research on Cancer (IARC) and are both linked to other respiratory diseases besides lung cancer, such as allergic asthma and chronic obstructive pulmonary disease (1,2). The mechanisms involved in lung carcinogenesis and the precise identity of the critical carcinogenic components in ambient air and diesel particulate matter (PM) are still unclear. To evaluate the health risks posed by these complex mixtures, understanding their mode(s) of action is crucial for accurate risk assessment. It is only modes and mechanisms that can assign causation of specific events to disease along an adverse outcome pathway from chemical exposure. Toxic chemicals absorbed to PM include polycyclic aromatic hydrocarbons (PAHs) as well as nitrated PAHs (nitro-PAHs), which require intracellular metabolic activation in order to exert their carcinogenic properties through binding to DNA and induction of mutations (3–7).

**Figure 1.**
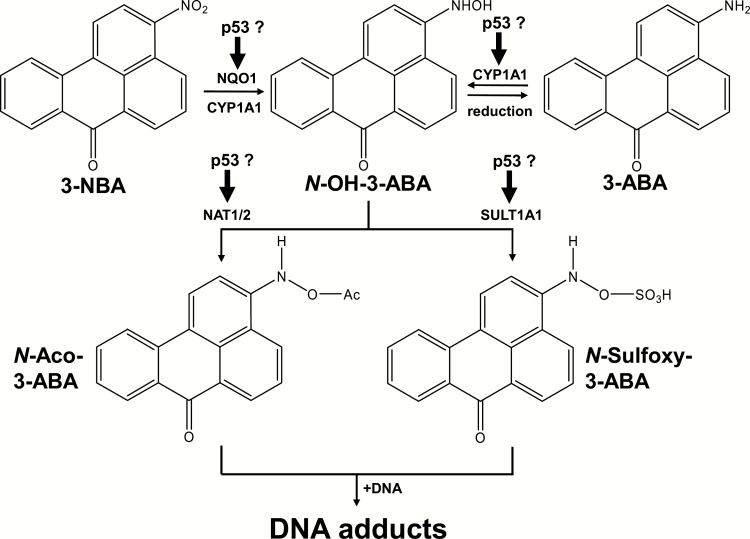
Possible impact of p53 on the major pathways of metabolic activation and DNA adduct formation of 3-NBA and 3-ABA. See text for details. Ac, –COCH_3_.

One of the nitro-PAHs present in diesel exhaust is the nitro-ketone 3-nitrobenzanthrone (3-NBA, 3-nitro-7*H*-benz[*de*]anthracen-7-one; Figure 1) (8). 3-NBA exhibits extremely high mutagenic activity *in vitro* and produces lung tumours in rats after intratracheal instillation (9). It has been classified as a possible human carcinogen (Group 2B) by IARC (1). The metabolic activation of 3-NBA to *N*-hydroxy-3-aminobenzanthrone (*N*-OH-3-ABA; Figure 1) is primarily catalysed by NAD(P)H:quinone oxidoreductase 1 (NQO1), which leads to the formation of reactive nitrenium/carbenium ions capable of reacting with DNA (10,11). *N*-OH-3-ABA can be further activated by acetylation or sulphation catalysed by *N*,*O*-acetyltransferases (NATs) and sulphotransferases (SULTs) forming highly reactive *N*-acetoxy- or *N*-sulphoxy esters (10,12–14). The main reductase metabolite of 3-NBA, 3-aminobenzanthrone (3-ABA; Figure 1) (15–17), has been identified in the urine of workers occupationally exposed to diesel exhaust (18). *N*-oxidation of 3-ABA catalysed by cytochrome P450 (CYP) enzymes (e.g. CYP1A1) also leads to the formation of *N*-OH-3-ABA (19,20). The major DNA adducts formed by 3-NBA both *in vitro* and *in vivo* after its metabolic activation by reduction of the nitro group are 2-(2ʹ-deoxyguanosin-8-yl)-3-aminobenzanthrone (dG-*N*^2^-3-ABA) and *N*-(2ʹ-deoxyguanosin-8-yl)-3-aminobenzanthrone (dG-C8-*N*-3-ABA) (21,22).

The *TP53* tumour suppressor gene, which encodes the protein p53, is one of the most important cancer genes (23–27). In response to cellular stress induced by various types of DNA damage, p53 maintains genomic integrity by delaying DNA synthesis or cell division to allow DNA repair, or inducing apoptosis (28). Disruption of the normal p53 response by *TP53* mutation leads to an increased risk of tumour development. *TP53* is mutated in over 50% of human tumours and various environmental carcinogens have been associated with characteristic mutational signatures in *TP53* (26,27). In addition to its role in the DNA damage response, p53 has also been found to regulate metabolic pathways such as glycolysis and oxidative phosphorylation thereby linking p53 not only to cancer but also to other diseases such as diabetes and obesity, and to other physiological processes such as ageing (29).

It has been observed that abrogation of p53 activity by knockout or knockdown of *TP53* in human cells affects carcinogen activation *in vitro* (23,30,31). We found that DNA adduct formation by the PAH benzo[*a*]pyrene (BaP) was significantly diminished in cells with altered cellular p53 function and that the observed DNA damage correlated with *CYP1A1* expression (23). Results indicated that BaP-induced CYP1A1 expression is regulated through p53 binding to a p53 response element in the regulatory region of *CYP1A1*, thereby enhancing its transcription (23). Similarly, a role for p53 in the CYP1A1-mediated metabolism of BaP was found *in vivo* in mice, although the mechanism involved in the expression of *CYP1A1* is different as lack of p53 function enhances BaP-DNA adduct formation *in vivo* (24). These studies reveal a new function of p53 in xenobiotic metabolism.

To evaluate the impact of the cellular *TP53* status on the metabolic activation of 3-NBA and its reduction metabolites *N*-OH-3-ABA and 3-ABA (Figure 1), we used a panel of isogenic human cell lines with differing *TP53* status, expressing either wild-type (WT) p53 [*TP53(+/+*)], heterozygous p53 [*TP53(+/−*)] or mutant p53 [*TP53(R248W/+*) or *TP53(R248W/−*)], or having a complete knockout of p53 [*TP53(−/−*)] (32). DNA adduct formation was determined by ^32^P-postlabelling. Expression of xenobiotic-metabolising enzymes (XMEs) involved in 3-NBA and 3-ABA metabolism was analysed by western blotting and quantitative reverse transcription polymerase chain reaction (RT-qPCR).

## Materials and Methods

### Carcinogens

As described previously, 3-NBA (CAS number 17117-34-9) (12) and 3-ABA (13) were prepared. The authenticity of 3-NBA and 3-ABA was confirmed by ultraviolet–visible spectroscopy, electrospray mass spectrometry and high-field proton nuclear magnetic resonance spectroscopy. *N*-OH-3-ABA was prepared as reported (20).

### Cell culture and chemical treatment

Through targeted homologous recombination, a panel of isogenic HCT116 human colorectal carcinoma cell lines has been developed that differ only with respect to their endogenous *TP53* status. *TP53(+/+*), *TP53(+/−*), *TP53(R248W/+*), *TP53(R248W/−*) and *TP53(−/−*) cells (32) were kindly provided by Prof. Bert Vogelstein, Johns Hopkins University School of Medicine, Baltimore, MD. The R248W mutation is found in some patients with Li-Fraumeni syndrome and leads to substitution of arginine for tryptophan, which results in modulated DNA binding capacity of the corresponding p53 protein product.

HCT116 cells were grown as adherent monolayers in complete growth medium: Dulbecco’s modified Eagle’s medium (#21885-025, Invitrogen) with 10% foetal bovine serum (#10106, Invitrogen), supplemented with 100 units penicillin and 100 µg streptomycin per millilitre. Cells were cultured at 37°C in 5% CO_2_ and passaged before the cells surpassed 80% confluence. For treatment, cells were seeded at 3 × 10^4^ cells/cm^2^, grown for 48 h and subsequently exposed for up to 48 h to a range of concentrations (0.5‒10 µM) of 3-NBA, 3-ABA and *N*-OH-3-ABA in order to find the optimal conditions for subsequent experiments, or solvent dimethyl sulphoxide (DMSO) as a control. The DMSO concentration was always kept at ≤0.5% of the total culture medium volume. Cells were harvested by trypsinisation and washed with phosphate-buffered saline (PBS).

### Cell viability by crystal violet staining

Cell viability after treatment with 3-NBA, 3-ABA and *N*-OH-3-ABA was investigated using a crystal violet staining assay. Briefly, the crystal violet (4-[(4-dimethylaminophenyl)-phenyl-methyl]-*N*,*N*-dimethylaniline) method is a colorimetric assay for measuring adherent cells, and staining of cells occurs through binding of the dye to DNA. Cells were seeded on 96-well plates, grown for 48 h and treated with various concentrations of the test compounds or DMSO as control as described earlier. At least six wells were used for testing each concentration. After treatment, the culture medium was removed, plates were washed gently with PBS before cells were fixed and stained with a 0.1% (w/v) crystal violet solution in 10% ethanol for 15 min. After staining, the extracellular dye was removed by rinsing the wells with PBS twice. Plates were dried before crystal violet was solubilised again in 50% ethanol, and a plate reader (Synergy HT; Biotek, UK) was used to record absorbance at 595 nm. Readings of DMSO-exposed cells were set to represent 100% viability, and results for each test compound were expressed as percentage of these controls. Experiments were performed in triplicate.

### DNA adduct analysis by ^32^P-postlabelling

Cells were seeded in 75-cm^2^ flasks and treated with the test compound or DMSO as control for up to 48 h as described earlier. DNA was isolated from cells using a standard phenol/chloroform extraction method. The butanol extraction enrichment version of the thin-layer chromatography (TLC) ^32^P-postlabelling assay was used to measure DNA adduct formation (33). The procedure was essentially as described previously (12) with minor modifications. Briefly, DNA samples (4 µg) were digested with micrococcal nuclease (288 mU: Sigma) and calf spleen phosphodiesterase (1.2 mU; MP Biomedical) and then enriched and labelled with 50 µCi γ-^32^P-ATP (Hartman Analytic, Germany) as reported. Solvent conditions for the separation of DNA adducts were as follows: D1, 1.0 M sodium phosphate, pH 6.0; D3, 4 M lithium formate, 7 M urea, pH 3.5; and D4, 0.8 M LiCl, 0.5 M Tris, 8.5 M urea, pH 8.0. After chromatography, TLC sheets were scanned using a Packard Instant Imager (Dowers Grove, IL, USA) and DNA adduct levels [relative adduct labelling (RAL)] were calculated from the adduct cpm, the specific activity of [γ-^32^P]ATP and the amount of DNA (pmol of DNA-P) used.

### Western blot analysis

Cells were seeded in 25-cm^2^ flasks and treated with the test compound or DMSO as control for 48 h as described earlier. Whole cell lysates were prepared as reported previously (23). Equal amounts of protein (10–20 µg) were separated by sodium dodecyl sulphate–polyacrylamide gel electrophoresis using 4–12% Bis-Tris gradient and western blotted as previously reported (34). After blocking in 3% nonfat milk (dissolved in PBS with 0.2% Tween-20), blots were incubated overnight (over 2 nights for *CYP1A1*) at 4°C with primary antibodies or antiserum diluted in blocking solution. The following primary antibodies and dilutions were used: anti-p53 1:2000 (Ab-6, Calbiochem), anti-p21 (CDKN1A) 1:2000 (556431, BD Pharmingen) and 1:10 000 anti-NQO1 (ab34173, Abcam). Anti-CYP1A1 raised in rabbits against purified human recombinant *CYP1A1* was a generous gift from Prof. F. Peter Guengerich (Vanderbilt University, USA) and was diluted 1:4000. Anti-SULT1A1/3 and anti-NAT1/2 were kindly provided by Prof. Hansruedi Glatt (German Institute of Human Nutrition, Nuthetal, Germany) and used at dilutions of 1:5000 and 1;10 000, respectively. These antisera were raised in rabbits against bacterial inclusion bodies of human SULT1A or NAT2 (35,36) and were shown to exhibit some cross-reactivity detecting human SULT1A1 and SULT1A3, or NAT1 and NAT2 (37). The antibody to detect GAPDH 1:25 000 (MAB374, Chemicon) was used as loading control. The secondary horseradish peroxidase-linked antibodies were anti-mouse (170–5047; 1:10 000)and anti-rabbit (170–5046; 1:10 000) from Bio-Rad. Visualisation of bands was accomplished using the enhanced chemiluminescent SuperSignal West Pico detection reagent according to the manufacturer’s instructions (Pierce, USA) and exposing the membranes to film. Incubations were carried out at least in duplicate.

### Gene expression analysis

Cells were seeded in 25-cm^2^ flasks and treated with the test compound or DMSO as control for 24 h as described earlier. RNA was isolated and reverse transcribed into cDNA as reported previously (23). Relative quantitation of *NQO1* and *CYP1A1* mRNA expression was performed using fluorescent RT-qPCR with the ABI PRISM 7500HT Fast Sequence Detection System (Applied Biosystems, UK) (23). *NQO1* and *CYP1A1* expression was detected using TaqMan^®^ gene expression primers and probes (*NQO1*-Hs02512143_s1 and *CYP1A1*-Hs00153120_m1) with GAPDH as endogenous control (*GAPDH*-Hs02758991_g1). Relative gene expression was calculated using the comparative threshold cycle (*C*_T_) method.

## Results

### Cell viability after exposure to 3-NBA and its metabolites

**Figure 2.**
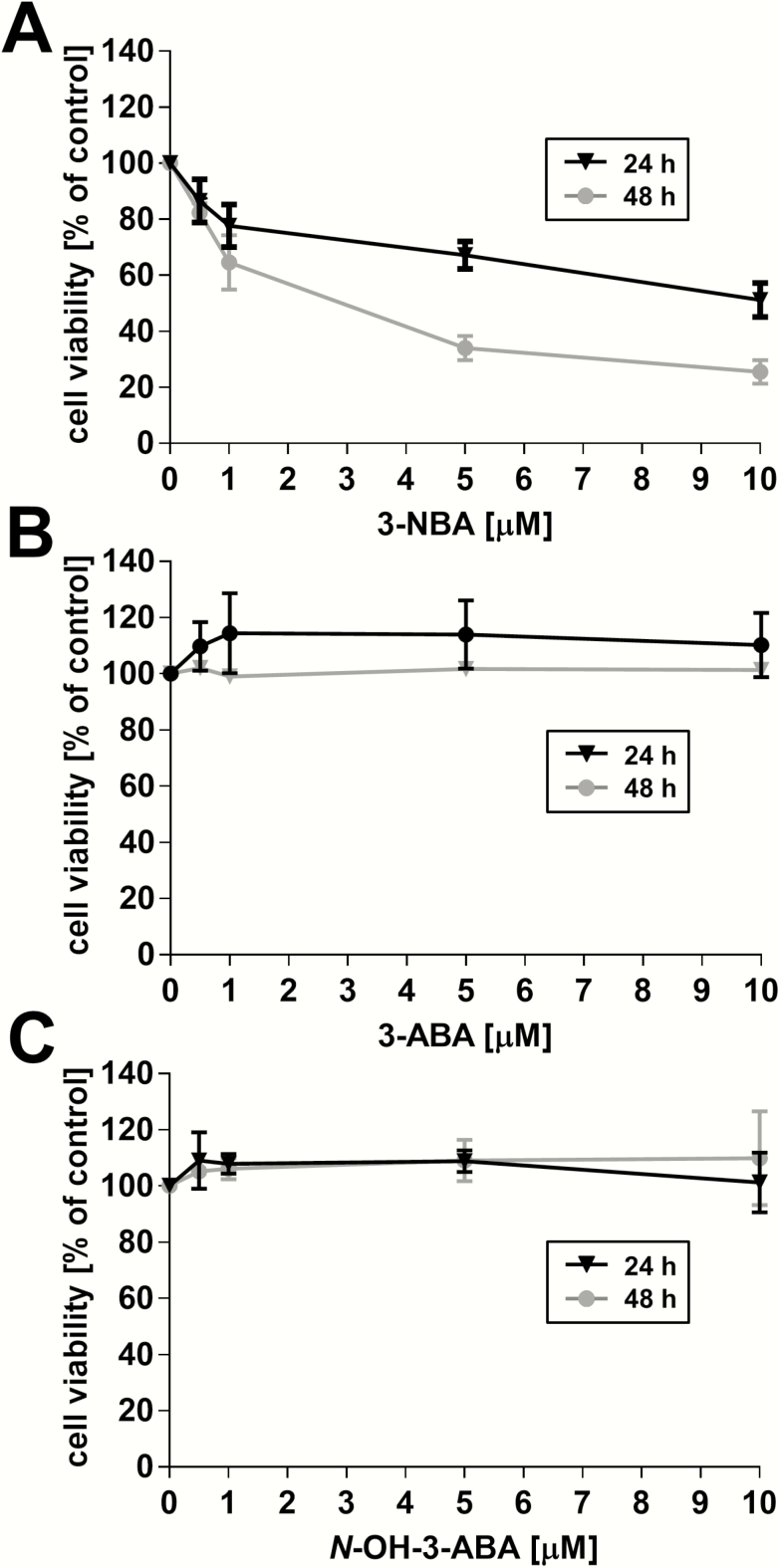
Effect of 3-NBA, 3-ABA and *N*-OH-3-ABA on cell viability in HCT116 *TP53(+/+*) cells. Cells were exposed to 0.5, 1, 5 and 10 µM of the test compounds for 24 h (black line) or 48 h (grey line). Values are means ± SD of two separate incubations with two biological samples each (*n* = 4).

At first, the effect of the test compounds on the viability of HCT116 *TP53(+/+*) cells was analysed by crystal violet staining to guide concentrations to be tested for each compound in further experiments in comparison with cells having altered *TP53* expression. HCT116 *TP53(+/+*) cells were subjected to treatment with 0.5, 1, 5 and 10 µM of 3-NBA, 3-ABA or *N*-OH-3-ABA, or with solvent DMSO only (control) for 24 and 48 h (Figure 2). After exposure to 3-NBA, *TP53(+/+*) cells exhibited a concentration-dependent decrease in cell viability, which was far more pronounced after 48 h compared to 24 h exposure. At the highest 3-NBA concentration (10 µM), viability decreased to ∼50% after 24 h and to ∼25% after 48 h in comparison to controls (Figure 2A). Treatment with 3-ABA and *N*-OH-3-ABA did not show any significant effect on cell viability at the used concentrations and time points (Figure 2B and C).

### DNA adduct formation after exposure to 3-NBA and its metabolites

**Figure 3.**
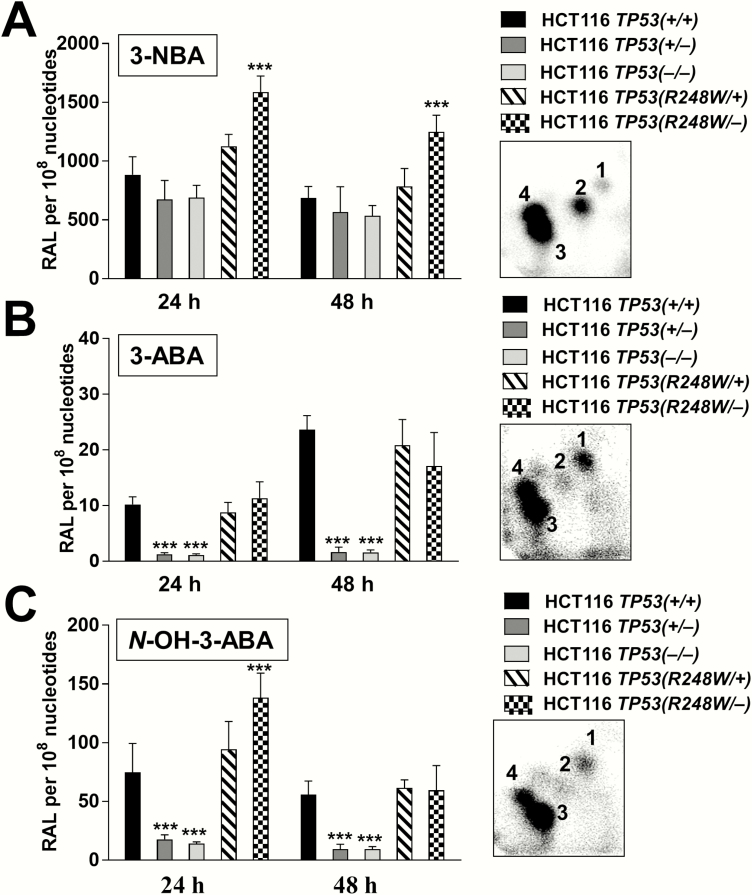
DNA adduct levels in isogenic HCT116 cells exposed to 3-NBA (A), 3-ABA (B) and *N*-OH-3-ABA (C) for 24 or 48 h. Cells were treated with 1 µM 3-NBA, 10 µM 3-ABA and 1 µM *N*-OH-3-ABA and analysed by ^32^P-postlabelling. Values are means ± SD of two separate incubations with two independent samples each (*n* = 4). Statistical analysis was performed by one-way ANOVA followed by the Tukey *post hoc* test [***P < 0.001; different from HCT116 *TP53(+/+*) cells]. Insets: Representative autoradiographic profiles of DNA adducts formed in HCT116 cells after exposure; the origins, at the bottom left-hand corners, were cut off before imaging.

To determine nitro/amino-PAH-derived DNA adduct formation, HCT116 *TP53(+/+*), *TP53(+/−*), *TP53(−/−*), *TP53(R248W/+*) and *TP53(R248W/−*) cells were exposed to 1 µM 3-NBA, 10 µM 3-ABA or 1 µM *N*-OH-3-ABA for 24 and 48 h and their DNA was analysed by the ^32^P-postlabelling method (Figure 3). The test concentrations were selected based on the cell viability data obtained in HCT116 *TP53(+/+*) (see Figure 2). For 3-NBA and *N*-OH-3-ABA, treatment conditions were in accordance with a previous study using HCT116 *TP53(+/+*) cells (38); 3-ABA had not been tested previously. Treatment with 3-NBA, 3-ABA and *N*-OH-3-ABA resulted in the formation of the same four major adduct spots in all cell lines (see inserts to Figure 3), which corresponded to adducts formed *in vivo* (15,21,22,38). Three of these adducts were previously identified as 2-(2ʹ-deoxyadenosin-*N*^6^-yl)-3-aminobenzanthrone (dA-*N*^6^-3-ABA, spot 1), *N*-(2ʹ-deoxyguanosin-*N*^2^-yl)-3-aminobenzanthrone (dG-*N*^2^-3-ABA, spot 3) and *N*-(2ʹ-deoxyguanosin-8-yl)-3-aminobenzanthrone (dG-C8-*N*-3-ABA, spot 4) (21). The adduct formed at spot 2 has not been identified fully yet but is categorised as a dA adduct (22).

Of the three compounds, 3-NBA gave rise to the highest total levels of DNA adducts (up to ∼1500 adducts per 10^8^ nucleotides). Used at 1 µM, 3-NBA-induced DNA adduct levels were ~10-fold higher than *N*-OH-3-ABA (1 µM) and ~100-fold higher than 3-ABA (10 µM). DNA adduct levels resulting from 3-NBA treatment were similar in all cell lines except for *TP53(R248W/−*) cells, which showed significantly higher adduct levels at both time points. 3-NBA-derived DNA adduct levels decreased slightly from 24 to 48 h (Figure 3A), which may be linked to increased cytotoxicity at 48 h.

Similar adduct levels were observed in *TP53(+/+*), *TP53(R248W/+*) and *TP53(R248W/−*) cells after treatment with 3-ABA and they increased from 24 to 48 h [*TP53(+/+*): ~2-fold; *TP53(R248W/+*): ~1.3-fold; *TP53(R248W/−*): ~1.3-fold]. Compared with *TP53(+/+*) cells, adduct levels were significantly lower (~8-fold at 24 h and ~14-fold at 48 h) in *TP53(+/−*) and *TP53(−/−*) cells (Figure 3B).

After treatment with *N*-OH-3-ABA, *TP53(+/+*) and *TP53(R248W/+*) cells formed similar amounts of DNA adducts at both time points (Figure 3C). *TP53(R248W/−*) cells also formed similar adduct levels 48 h after treatment but significantly higher DNA adducts were detected at 24 h for this cell line. As seen for 3-ABA, DNA adduct levels were significantly lower in *TP53(+/−*) and *TP53(−/−*) cells compared to *TP53(+/+*) cells [i.e. *TP53(+/−*): 4-fold at 24 h and 6-fold at 48 h; *TP53(−/−*): 5-fold at 24 h, 6-fold at 48 h]. Generally, *N*-OH-3-ABA-derived DNA adduct levels decreased slightly from 24 to 48 h.

### Expression of DNA damage response proteins after exposure to 3-NBA and its metabolites

**Figure 4.**
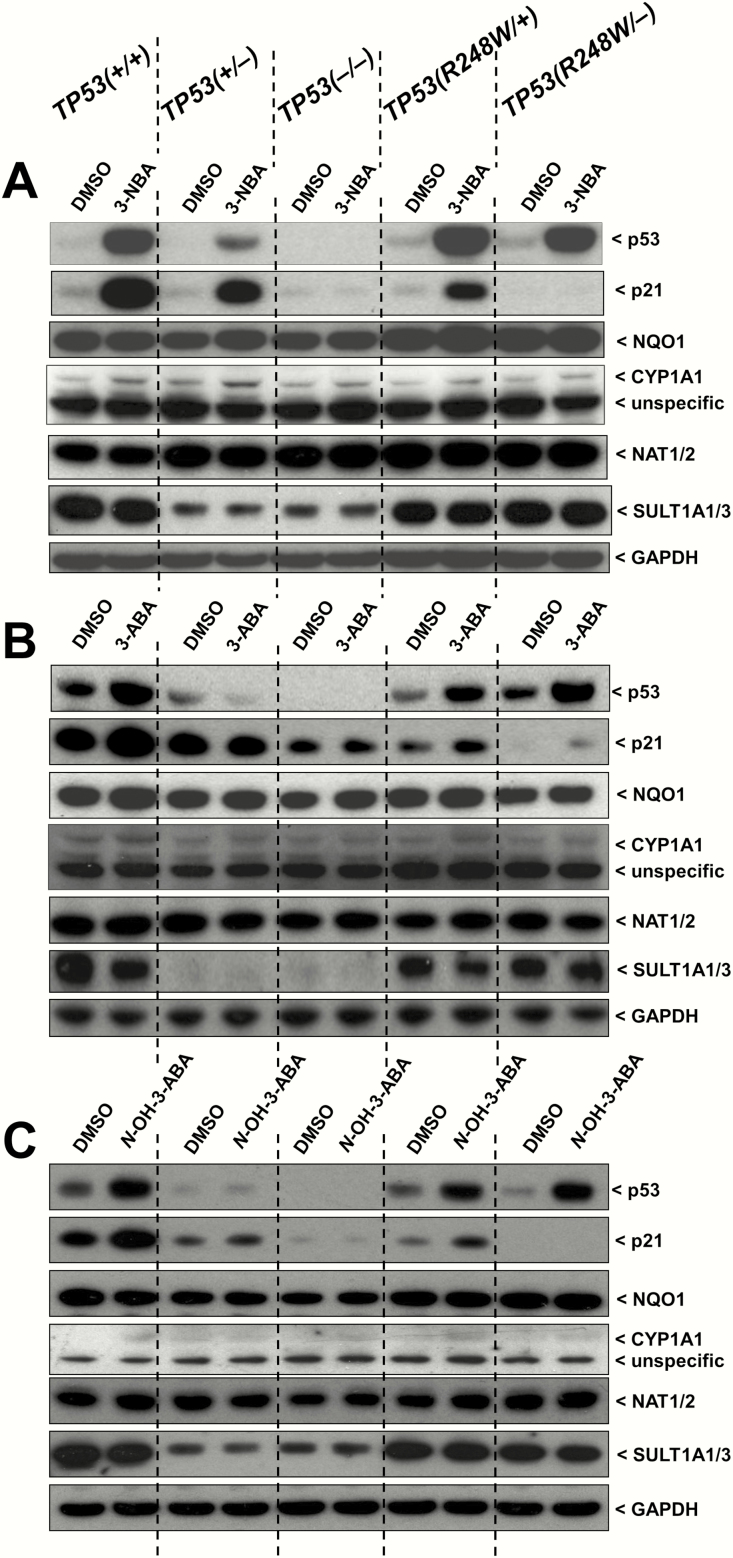
Western blot analysis of p53, p21 (CDKN1A), NQO1, CYP1A1, NAT1/2 and SULT1A1/3 protein expression in isogenic HCT116 cells after exposure to 3-NBA (A), 3-ABA (B) and *N*-OH-3-ABA (C) for 48 h. Cells were treated with 1 µM 3-NBA, 10 µM 3-ABA and 1 µM *N*-OH-3-ABA. GAPDH protein expression was used as a loading control. Representative images of the Western blotting are shown; at least duplicate analysis was performed from independent experiments.

HCT116 *TP53(+/+*), *TP53(+/−*), *TP53(−/−*), *TP53(R248W/+*) and *TP53(R248W/−*) cells were treated with 1 µM 3-NBA, 10 µM 3-ABA, 1 µM *N*-OH-3-ABA or DMSO alone (control) for 48 h and whole cell lysates were analysed for the expression of p53 and p21 by western blotting. Both proteins have been shown to be sensitive markers to assess DNA damage response in HCT116 cells (23,39). As seen in Figure 4, treatment with 3-NBA resulted in strong induction of p53 in *TP53(+/+*), *TP53(R248W/+*) and *TP53(R248W/−*) cells whereas 3-ABA and *N*-OH-3-ABA induced p53 to a lesser extent in these cell lines. In *TP53(+/−*) cells, basal levels and induction of p53 were strongly decreased and no p53 was detectable in *TP53(−/−*) cells. Induction of p21 was strongest after treatment with 3-NBA whereas 3-ABA and *N*-OH-3-ABA led to only moderate changes compared with controls. Induced and basal p21 expression was highest in *TP53(+/+*), followed by *TP53(+/−*) and *TP53(R248/+*) cells and greatly decreased in *TP53(−/−*) and *TP53(R248/−*) cells.

### Expression of XMEs after exposure to 3-NBA and its metabolites

**Figure 5.**
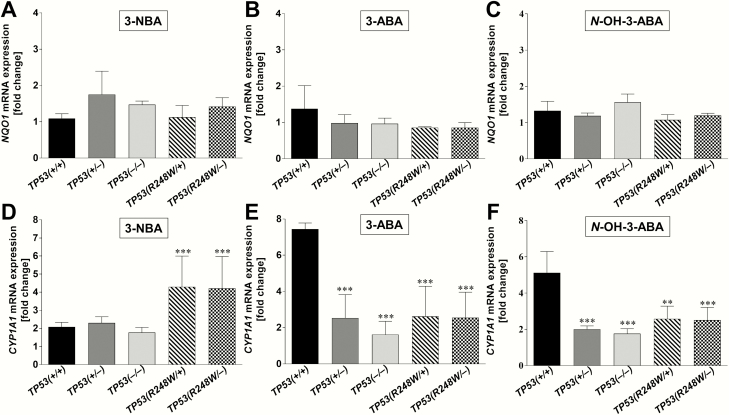
Change in gene expression of *NQO1* (upper panels) and *CYP1A1* (lower panels) in isogenic HCT116 cells after exposure to 3-NBA, 3-ABA and *N*-OH-3-ABA. Cells were exposed to 1 µM 3-NBA (A + D), 10 µM 3-ABA (B + E) and 1 µM *N*-OH-3-ABA (C + F) for 24 h. In addition, control samples for each cell line were treated with DMSO before total RNA was extracted, and the mRNA levels of the indicated genes were analysed by qRT-PCR. Values are means ± SD of three incubations; each sample was determined by three separate analyses. Each bar represents the fold change in expression caused by exposure to the respective compound (3-NBA, 3-ABA or *N*-OH-3-ABA) of each cell line and was calculated using the formula 2^−ΔΔCT^. Statistical analysis was performed by one-way ANOVA followed by the Tukey *post hoc* test [**P < 0.01, ***P < 0.001; different from HCT116 *TP53(+/+*) cells].

It has been shown that 3-NBA is predominantly activated by NQO1 (10,11). Using Western blotting, we determined NQO1 protein expression in whole cell lysates isolated from HCT116 *TP53(+/+*), *TP53(+/−*), *TP53(−/−*), *TP53(R248W/+*) and *TP53(R248W/−*) cells treated with 1 µM 3-NBA, 10 µM 3-ABA, 1 µM *N*-OH-3-ABA or DMSO alone (control) for 48 h (Figure 4). Expression of NQO1 protein was treatment independent and similar in all cell lines. These findings were in line with the RT-qPCR data in HCT116 cells exposed under the same experimental conditions for 24 h; no significant differences in *NQO1* gene expression were detectable between the cell lines after exposure to any of the three compounds (Figure 5A–C).

3-ABA is activated by CYP1A1 (16). Two bands were detected on the western blot for CYP1A1; the top band is the correct molecular weight (58 kDa). In addition, previous experiments have shown that the top band increases with higher BaP concentrations used and also that it diminishes when BaP-treated cells have been transfected with *CYP1A1* siRNA (Kucab and Arlt, unpublished data). Thus, the lower band is assumed to be nonspecific. The antibody has been shown to be sensitive to detect human CYP1A1 protein in cultured human cells (including HCT116) exposed to BaP (23,39–41). However, in this study CYP1A1 protein expression after 3-NBA, 3-ABA and *N*-OH-ABA exposure was weak or hardly detectable (Figure 4). These findings could indicate that 3-NBA, 3-ABA and *N*-OH-ABA, in contrast to BaP, do not or only weakly induce CYP1A1 expression in HCT116 cells. However, they may also be due to the insensitivity of the antibody to detect low amounts of CYP1A1 protein in whole cell lysates. As CYP1A1 protein expression in HCT116 cells after 3-NBA, 3-ABA and *N*-OH-ABA exposure was low and close to background, no solid conclusions could be drawn on the impact of p53 function. As a more sensitive measure, we therefore measured and quantified *CYP1A1* gene expression by RT-qPCR (Figure 5D–F). For 3-NBA treatment, the increase in *CYP1A1* expression was significantly higher (~2-fold) in *TP53(R248W/+*) and *TP53(R248W/−*) cells compared with the other cell lines (Figure 5D). It is noteworthy that in *TP53(R248W/−*) cells increased *CYP1A1* expression correlated with higher DNA adduct levels (see Figure 3A). In *TP53(+/+*), *TP53(+/−*) and *TP53(−/−*) cells, 3-NBA treatment induced *CYP1A1* to a similar extent, which correlated with similar adduct levels. These results suggest that mutant-R248W-p53 but not WT-p53 impacts on 3-NBA-mediated *CYP1A1* induction. In contrast, exposure to 3-ABA and *N*-OH-3-ABA led to significantly higher *CYP1A1* mRNA levels in *TP53(+/+*) cells compared to the other cell lines, pointing towards a WT-p53-dependent impact on the CYP1A1-mediated bioactivation of these compounds. Overall, *CYP1A1* expression was altered to a greater extent than *NQO1*, suggesting a greater role of the former in modulating the metabolism of these compounds in these cells.

*N*-OH-3-ABA can be further activated by SULTs, particularly SULT1A1 (10,12,13). The SULT1A antiserum used is able to detect SULT1A1 and SULT1A3 protein (37). As both SULT1A enzymes show cross-reactivity and the same electrophoretic mobility expression, they were analysed as a group rather than as individual proteins. SULT1A1/3 protein expression was not affected by treatment with any of the compounds, but basal levels were highest in *TP53(+/+*), *TP53(R248W/+*) and *TP53(R248W/−*) cells and were substantially lower in *TP53(−/−*) and *TP53(+/−*) cells (Figure 4).

NAT1 and NAT2 have also been shown to enhance the genotoxicity of both 3-NBA and 3-ABA (10,12,13). The NAT1/2 antiserum used has previously shown cross-reactivity towards NAT1 and NAT2 (37) and expression of both was analysed as a group because separation of the two bands proved difficult (Figure 4). Expression of NAT1/2 protein was treatment independent and similar in all cell lines.

## Discussion

Our study aimed to evaluate the impact of p53 function on the metabolic activation of the carcinogenic air pollutant 3-NBA and its reduction metabolites *N*-OH-3-ABA and 3-ABA. Because no human lung cell lines with disrupted *TP53* were available, we used isogenic human colorectal HCT116 differing only with respect to their *TP53* status; these cells were created by targeted homologous recombination. They have been proven to be a valuable tool to investigate cellular responses associated with the *TP53* network including carcinogen metabolism (23,30,31,39,42–44). In addition, previous studies have used human HCT116 cells to investigate the genotoxicity of 3-NBA and/or *N*-OH-3-ABA (30,31,38).

Similar 3-NBA-derived DNA adduct levels in the HCT116 *TP53(+/+*), *TP53(+/−*) and *TP53(−/−*) cell lines suggest a WT-*TP53*-independent metabolic activation of this compound. These adduct results for 3-NBA in the *TP53(+/+*) and *TP53(−/−*) cell lines are in line with earlier findings (30,31) when tested at a higher concentration (i.e. 5 µM). Because bioactivation of 3-NBA by simple nitroreduction is primarily catalysed by NQO1, rather than CYP enzymes (8,11,45), these results indicate that NQO1 expression is not influenced by *TP53* status. This corresponds with the observation that no impact of WT-p53 status was seen on the induction of NQO1 at either the protein or mRNA level. Similarly WT-p53 status had no effect on *CYP1A1* mRNA expression.

Nucleotide excision repair (NER) is the main DNA repair pathway for many bulky DNA adducts such as those derived from PAHs such as BaP, and p53-dependent pathways affecting global NER have been identified (26). Using a modified comet assay, we previously phenotypically assessed the NER capacity of the different HCT116 cells and found that all HCT116 cell lines used had the same NER capacity (23). Other studies have suggested that 3-NBA-DNA adducts may not be recognised by NER, explaining the high mutagenic potency of 3-NBA (27). Collectively these findings indicate that differences in DNA adduct formation in HCT116 cell lines exposed to 3-NBA and its metabolites are solely related to differences in their bioactivation and not to p53-dependent differences in DNA repair.

DNA adduct levels formed after incubations with the metabolite 3-ABA were significantly lower in *TP53(+/−*) and *TP53(−/−*) cell lines compared with *TP53(+/+*) cells. As bioactivation (i.e. *N*-oxidation) of 3-ABA to *N*-OH-3-ABA is catalysed by CYP1A1 (15,16,46,47), these results can be explained by diminished induction of *CYP1A1* mRNA in these cell lines compared with *TP53(+/+*) cells (Figure 1). Previous studies have also shown that 3-ABA is able of inducing CYP1A1 expression, thereby enhancing its own metabolic activation (48,49). Taking the results of this study into account, these findings indicate an influence of WT-p53 on the CYP1A1-mediated bioactivation of 3-ABA.

Both 3-NBA and 3-ABA share the same intermediate *N*-OH-3-ABA, which is capable of reacting with DNA to form adducts (20,38). The genotoxicity of *N*-OH-3-ABA can be enhanced by SULTs or NATs, leading to reactive *N*-acetoxy or *N*-sulphoxy esters (10,12,14,19). *N*-OH-3-ABA-induced DNA adducts were lower in *TP53(+/−*) and *TP53(−/−*) cells than in *TP53(+/+*) cells, which corresponded with lower SULT1A1/3 protein expression in *TP53(+/−*) and *TP53(−/−*) cells, whereas NAT1/2 protein expression was similar in all cell lines. These results suggest that the SULT-mediated bioactivation of *N*-OH-3-ABA seems to be dependent on WT-p53.

As *N*-OH-3-ABA is a metabolite of both 3-NBA and 3-ABA, the impact of WT-p53 on SULT expression influencing DNA binding by *N*-OH-3-ABA might also contribute to 3-NBA- or 3-ABA-DNA adduct formation. However, DNA adduct formation by 3-NBA was not significantly different in *TP53(+/−*), *TP53(−/−*) and *TP53(+/+*) cells suggesting that in HCT116 cells the rate-limiting step of the bioactivation pathway of 3-NBA is the conversion of 3-NBA to *N*-OH-3-ABA. In contrast, because p53 impacts on the expression of both SULT1A1/3 and CYP1A1 after 3-ABA incubation, it is not clear which is the rate-limiting step in the metabolic activation of 3-ABA.

It has been shown that partial inactivation of tumour suppressor genes such as *TP53* can contribute to carcinogenesis, suggesting that one copy of *TP53* is not sufficient to maintain a functional WT condition (haploinsufficiency). Loss of a single copy of *TP53* can give rise to a phenotype intermediate to that occurring after complete loss of the gene (50,51). Therefore, it could have been expected that HCT116 *TP53(+/−*) cells would generate an intermediate response to those observed in *TP53(−/−*) and *TP53(+/+*) cells. However, the results obtained for 3-ABA and *N*-OH-3-ABA suggest that in HCT116 *TP53(+/−*) cells, the remaining WT allele is not sufficient for normal cellular function, which was also indicated in another study using these cells (52). Similar results have been observed in HCT116 *TP53(+/−*) cells treated with PAHs such as BaP, dibenz[*a,h*]anthracene and dibenzo[*a,l*]pyrene (23). An explanation could be that transcriptional activation by p53 is dependent on its tetrameric structure and that the 50% reduction in protein dosage results in a disproportionate reduction in active tetramer concentrations (53).

More than 50% of human tumours carry a mutation in *TP53* and some mutation patterns and spectra in *TP53* in human tumours have been linked to specific environmental exposures (26,28). It has also been shown that 3-NBA induces characteristic mutations in *TP53* using the human *TP53* knock-in (Hupki) mouse model (27). Mutations in *TP53* can not only abrogate the tumour suppressor functions of WT-p53 (loss-of-function mutation) but can also equip the mutant protein with new activities (gain-of-function mutation), which can contribute to various stages of tumour progression (54). As shown in the IARC TP53 Database (http://p53.iarc.fr; version R18), codon 248 is the most frequently mutated codon in *TP53* in human tumours. The exchange of a native arginine for tryptophan in p53’s DNA-binding region (R248W) induces conformational changes and has been shown to abolish the tumour suppressive activity of p53 (55). Thus, studying the response of cells with this mutation (R248W) in *TP53* to environmental carcinogens can give insights into how mutations contribute to carcinogenesis.

3-ABA-DNA adduct levels in both cell lines that carried mutated p53 were similar to that in *TP53(+/+*) cells. Whereas the higher levels of 3-ABA-DNA adducts in *TP53(+/+*) compared with *TP53(+/−*) and *TP53(−/−*) cells could be explained by higher *CYP1A1* expression in the former, this could not be seen for *TP53(R248W/+*) or *TP53(R248W/−*). Rather they could be interpreted as the result of higher SULT1A1/3 expression in these cell lines.

It has been shown that under anaerobic conditions, CYP1A1 is also capable of catalysing the nitroreduction of 3-NBA (19,56) although this reaction is otherwise primarily catalysed by NQO1 (11). In *TP53(R248W/−*) cells, significantly higher 3-NBA-DNA adducts corresponded with a significantly greater induction of *CYP1A1*, but although *CYP1A1* was induced to a similar extent in *TP53(R248W/+*) cells, it did not result in the same amount of 3-NBA-DNA adducts. It can be speculated that CYP1A1 expression impacts on 3-NBA-DNA adduct formation in these cells. However, why the significant difference in *CYP1A1* expression in both cell lines that have mutant p53 results in significantly higher adduct levels only in *TP53(R248W/−*) cells and not in *TP53(R248W/+*) cells remains unclear. Similarly, as SULT1A1/3 and NAT1/2 expression was equally high in *TP53*(R248W/*−*), *TP53(R248W/+*) and *TP53(+/+*) cells, the contrasting 3-NBA-DNA adduct levels in *TP53(R248W/−*) cells seem not to be attributable to SULT-mediated bioactivation of this compound. These findings indicate that the influence of mutated p53 on the regulation of XMEs might be more complex. Furthermore, they also suggest that studying the impact of p53, whether WT or mutated, on carcinogen metabolism is a challenge if p53 function impacts on multiple XMEs.

The same phenomenon was observed after treatment with *N*-OH-3-ABA. Although *TP53(+/+*), *TP53(R248W/+*) and *TP53(R248W/−*) cells displayed similar SULT1A1/3 and NAT1/2 protein expression, *N*-OH-3-ABA-DNA adduct levels were significantly higher in *TP53(R248W/−*) cells, showing that the mutant-p53 mechanism of action differs from that of WT-p53. Thus, these findings demonstrate a gain-of-function for the mutated R248W-p53 protein. Similarly, acquisition of new functions for this mutant has been shown before in the HCT116 *TP53(R248W/−*) cell line (57) and in the Hupki mouse model (55). In this study, the acquired function might be exerted through activation of other XMEs.

Another enzyme that has been shown to activate both 3-NBA and 3-ABA is CYP1A2 (56,58). Although *CYP1A2* expression was not determined in this study, it is known to be restricted predominantly to the liver (59), thus its contribution to the bioactivation of 3-NBA and, particularly, of 3-ABA in the colorectal HCT116 cells used here would be expected to be negligible. It is noteworthy that treatment of HCT116 *TP53(+/+*) cells with 2-amino-1-methyl-6-phenylimidazo[4,5-*b*]pyridine (PhIP), which is mainly activated by CYP1A2, did not lead to detectable DNA adduct formation even at high concentrations of up to 200 µM (Wohak *et al*., unpublished data). Conversely, p53-dependent PhIP-DNA adduct formation has been demonstrated in a *Trp53* mouse model *in vivo* (25). *N*-OH-3-ABA can also be conjugated by another enzyme, SULT1A2 (10,12). Tissue distribution of this SULT is restricted mainly to the liver and bladder (60), although low amounts have been detected in another human colon carcinoma cell line (Caco-2) (61) and in some human colon samples (36). Furthermore, the microsomal enzyme NADPH:P450 oxidoreductase (POR), which is the electron donor of CYP enzymes (62), is also capable of activating 3-NBA through simple nitroreduction in cell-free experimental systems (56). However, using the Hepatic Reductase Null mouse model, in which POR is specifically deleted in hepatocytes, it was shown that bioactivation of 3-NBA is dependent predominantly on cytosolic NQO1 rather than microsomal POR (10). Although the activation pathways of 3-NBA and its metabolites have been well studied (8,19), knowledge of the enzymes detoxifying 3-NBA is sparse. Thus it is possible that other XMEs that have not yet been investigated might influence the results obtained in the HCT116 model.

In both cases where a significant impact of the mutant p53 on adduct formation (3-NBA- and *N*-OH-3-ABA-derived adducts) was found in *TP53(R248W/−*) cells, *TP53(R248W/+*) cells generated DNA adduct levels intermediate between the *TP53(R248W/−*) and *TP53(+/+*) cell lines. This might indicate that this altered function of the R248W-p53 may be compensated for by a WT-p53 in the *TP53(R248W/+*) cell line.

Although there are previous reports that NATs, especially NAT2, may be more important than SULTs in the bioactivation of 3-NBA (8,10,11), this study indicates that in HCT116 cell lines, the SULTs seem to have a greater impact.

Our past studies (23–25,30,31,39), the present one and those of others (63–66), also demonstrate that p53’s influence depends on the class of xenobiotic compound and thereby on the XMEs that mediate the bioactivation of the particular compounds. To form conclusions for the whole class of nitro-PAHs, this study will have to be widened to include other nitro-PAHs. Another nitroaromatic carcinogen that has been tested in HCT116 *TP53(+/+*) and *TP53(−/−*) cells is aristolochic acid I (AAI) (31), a potent human carcinogen linked to urothelial cancer (67,68). AAI is primarily activated by NQO1-catalysed nitroreduction, whereas CYP1A-mediated demethylation contributes to AAI detoxification (69). Previous studies have shown that AAI–DNA adduct formation was significantly higher in *TP53(+/+*) relative to *TP53(−/−*) cells and cellular *TP53* status may impact on CYP1A1-mediated AAI bioactivation (31,70).

## Conclusions

This study illustrates the impact of p53 function on the metabolic activation of 3-NBA and its metabolites 3-ABA and *N*-OH-3-ABA. Although WT-p53 had no influence on 3-NBA-derived DNA adduct formation, lower 3-ABA- and *N*-OH-ABA-derived DNA adducts levels in *TP53(+/−*) and *TP53(−/−*) cells than in *TP53(+/+*) cells corresponded with diminished expression of *CYP1A1* mRNA and SULT1A1/3 protein in these cells. Our results show that p53’s influence on carcinogen activation depends on the class of carcinogen and on the XMEs that mediate the bioactivation of the particular xenobiotic. The influence of p53 on regulating CYP1A1 and SULT1A1/3 expression is in line with other recent studies demonstrating this phenomenon (23–25,39). However, this is the first study highlighting the impact of p53 on SULT-mediated carcinogen metabolism in human cells. Because SULTs are involved in controlling the balance of many endogenous molecules such as steroids, sterols, thyroid hormones and catecholamines (60,71), our results may also suggest a role for p53 in endocrine signalling pathways. Our study also showed that mutant R248W-p53 protein function was similar to, or even exceeded, the ability of WT-p53 in activating 3-NBA and its metabolites, measured as DNA adducts, but it is possible that cells carrying a different *TP53* mutation to R248W may respond differently. Future studies could aim for a more comprehensive approach to examining the impact of distinct *TP53* mutations in isogenic cells for a comparative functional analysis of mutant p53 and carcinogen metabolism.

## Funding

Work at King’s College London was supported by Cancer Research UK (grant C313/A14329), the Wellcome Trust (grants 101126/Z/13/Z and 101126/B/13/Z) and the National Institute for Health Research Health Protection Research Unit (NIHR HPRU) in Health Impact of Environmental Hazards at King’s College London in partnership with Public Health England (PHE) and Imperial College London. L.E.W was supported by a PhD studentship from the Institute of Cancer Research. A-C.B was supported by a Leonardo da Vinci scholarship and the United Kingdom Environmental Mutagen Society (UKEMS). A.M.K was supported by a fellowship from the German Research Foundation (DFG).
